# Analyzing the value of monitoring duodenal mucosal perfusion using photoplethysmography

**DOI:** 10.1186/s13054-014-0561-6

**Published:** 2014-10-13

**Authors:** Mitchell P Fink

**Affiliations:** Departments of Surgery and Anesthesiology, David Geffen School of Medicine at UCLA, 10833 Le Conte Avenue, 72-160 CHS, Los Angeles, CA 90095 USA

## Abstract

Photoplethysmography (PPG) is a technique that permits noninvasive measurement of changes in the volume of tissues. A novel device uses PPG to assess changes in duodenal mucosal perfusion. When tested in septic piglets, data obtained using this device correlate with the blood lactate concentration and duodenal serosal microvascular blood flow as measured with a laser Doppler flowmeter. This new PPG-based approach for continuously monitoring gut mucosal perfusion warrants further development, leading to prospective clinical trials in patients.

In the previous issue of *Critical Care*, Jacquet-Lagrèze and colleagues from several institutions in Lyon, France, report results from a preclinical study of a novel perfusion monitoring device [1]. The study was carried out using anesthetized and mechanically ventilated piglets. Some of the animals were infused with a suspension of viable *Pseudomonas aeruginosa* to induce septic shock; the remaining (control) animals were not challenged with the Gram-negative bacteria preparation.

The novel monitoring device was developed by Advanced Perfusion Diagnostics [2], a biotechnology start-up company in Lyon. The device makes use of photoplethysmography (PPG) to assess changes in duodenal mucosal blood flow.

PPG and its applications in medicine have been well described in an excellent review article by Allen [3]. PPG uses light in the visible red and near-infrared regions of the spectrum to non-invasively assess changes in the volume of a specific region of tissue. PPG can be carried out using light that is transmitted through tissue or using light that is reflected by the tissue. Light-emitting diodes provide the light in present-day commercially available devices that employ PPG for medical applications, such as beat-to-beat monitoring of blood pressure.

The characteristic signal from a PPG device is a waveform. The dominant (peak and valley) aspect of the tracing is synchronized with the beating of the heart, and is usually called the alternating current (AC) component. The origins of the other components of the PPG signal remain to be completely elucidated, although important factors are recognized to be changes in circulating blood volume, respiration and vasomotor tone. The quasi-static component of the PPG signal is usually called the direct current (DC) component.

The device, which was evaluated by Jacquet-Lagrèze and colleagues, features a reflectance type of PPG device fitted onto the surface of a balloon located near the distal end of a small bore feeding tube. When the end of the feeding tube is advanced through the pylorus into the duodenum and the balloon is inflated, the PPG element presses against the mucosal surface of this portion of the intestine, allowing continuous monitoring of the AC and DC components of the waveform. In their studies using pigs, the research group from Lyon showed that the variations from baseline in both the AC and DC components of the duodenal mucosal PPG signal were significantly correlated with the variations from baseline in duodenal serosal microvascular blood flow measured using laser Doppler flowmetry. Importantly, the AC and DC components of the PPG signal also were significantly correlated with the blood lactate concentration. The findings from this preclinical validation study thus support the view that the PPG-based duodenal mucosal blood flow monitoring device provides meaningful data.

While the report by Jacquet-Lagrèze and colleagues should provide encouragement for the company that is developing the new monitoring device, a look back at the history of this field should temper their enthusiasm and ours. As pointed out by the authors, use of another gastrointestinal perfusion monitoring device – the gastric tonometer – was shown to significantly improve survival in a subset of critically ill patients [4]. But the widespread adoption of this device or even an improved version of it never happened, possibly because some clinical studies showed that the method failed to provide reliable information about splanchnic blood flow [5] or, more importantly, failed to provide added value compared with that obtained from routine measurements of arterial blood gases [6]. It seems most likely, however, that gastric tonometry fell out of favor because clinicians, when confronted with evidence for inadequate gut mucosal perfusion, were perplexed about the proper intervention(s), especially when other commonly used indices of perfusion – such as arterial blood pressure, mixed venous oxygen saturation, and cardiac output – were sending a different message.

Duodenal mucosal perfusion monitoring, using a cleverly designed PPG-based device, warrants further development, ultimately leading to prospective clinical trials. When these trials are designed, it will be important to pay careful attention to the intervention(s) that are triggered when the device indicates that intestinal mucosal perfusion is low.

## Abbreviations

AC: Alternating current
DC: Direct current
PPG: Photoplethysmography

## References

[1] Jacquet-Lagrèze M, Bonnet-Garin J-M, Allaouchiche B, Vassal O, Restagno D, Paquet C, Ayoub J-Y, Etienne J, Vandenesch F, Daulwader O, Junot S (2014). A new device for continuous assessment of gut perfusion: proof of concept on a porcine model of septic shock. Crit Care.

[2] **Advanced Perfusion Diagnostics** [www.apdiagnostics.net]

[3] Allen J (2007). Photoplethysmography and its application in clinical physiological measurement. Physiol Meas.

[4] Gutierrez G, Palizas F, Doglio G, Pusajo J, Wainsztein N, Klein F, Gallesio A, San Roman E, Pacin J, Dorfman B, Dubin A, Schiavi E, Shottender J, Jorge M, Giniger R (1992). Gastric intramucosal pH as a therapeutic index of tissue oxygenation in critically ill patients. Lancet.

[5] Uusaro A, Ruokonen E, Takala J (1995). Gastric mucosal pH does not reflect changes in splanchnic blood flow after cardiac surgery. Br J Anaesth.

[6] Boyd O, Mackay CJ, Lamb G, Bland JM, Grounds RM, Bennett ED (1993). Comparison of clinical information gained from routine blood-gas analysis and from gastric tonometry for intramural pH. Lancet.

